# X-ray diffraction imaging of metal–oxide epitaxial tunnel junctions made by optical lithography: use of focused and unfocused X-ray beams

**DOI:** 10.1107/S090904951204856X

**Published:** 2013-01-19

**Authors:** Cristian Mocuta, Antoine Barbier, Stefan Stanescu, Sylvia Matzen, Jean-Baptiste Moussy, Eric Ziegler

**Affiliations:** a Synchrotron SOLEIL, L’Orme des Merisiers, Saint-Aubin, BP-48, F-91192 Gif-sur-Yvette, France; b CEA-Saclay, DSM/IRAMIS/SPCSI, F-91191 Gif-sur-Yvette, France; c European Synchrotron Radiation Facility, 6 rue Jules Horowitz, BP-220, F-38043 Grenoble Cedex 9, France

**Keywords:** X-ray imaging with diffraction contrast, microbeam X-ray diffraction, raster microscopy, metal–oxide epitaxial tunnel junctions

## Abstract

X-ray diffraction techniques are used in imaging mode in order to characterize micrometre-sized objects. The samples used as models are metal–oxide tunnel junctions made by optical lithography, with lateral sizes ranging from 150 µm down to 10 µm and various shapes: discs, squares and rectangles. Two approaches are described and compared, both using diffraction contrast: full-field imaging (topography) and raster imaging (scanning probe) using a micrometre-sized focused X-ray beam. It is shown that the full-field image gives access to macroscopic distortions (*e.g.* sample bending), while the local distortions, at the micrometre scale (*e.g.* tilts of the crystalline planes in the vicinity of the junction edges), can be accurately characterized only using focused X-ray beams. These local defects are dependent on the junction shape and larger by one order of magnitude than the macroscopic curvature of the sample.

## Abstract 2

Metal-oxide tunnel junctions made by optical lithography were studied by X-ray diffraction (imaging approach with diffraction contrast) to access local distortions of the crystalline planes of the constituting layers. The effect of the size (10–150 µm) and shape (disc, square and rectangle) of the junction was investigated.

## 1. Introduction   

Imaging microscopy techniques are valuable tools providing important information about complex systems leading to an improved understanding of their physical properties. Well established techniques using local probe microscopy (atomic force microscopy, AFM; scanning tunnelling microscopy, STM; scanning electron microscopy, SEM; *etc*.) allow easy access of information at the sub-nanometre scale. Various contrast mechanisms (topography, chemical, *etc*.) can be exploited in order to obtain images of the samples. In the present paper we consider an approach using X-rays and diffraction contrast. In some cases it can become the technique of choice, giving access to new information, not otherwise available. It should thus be considered not as an alternative but more likely complementary to the above-mentioned microscopy techniques.

The advantages of local probe microscopy methods, already established, are undeniable. Very good resolution images of the sample surface can be obtained rather quickly, with details on the nanometre scale. Various contrasts, including chemical, can be used to generate a sample image. They do remain mostly surface-sensitive techniques, thus the sample needs to be mechanically prepared (thinned, cleaved, etched) if information from below the surface is needed (*e.g.* buried interfaces or layers). X-ray diffraction (XRD) using hard X-rays (energies in the 10 keV range) has the advantage of probing deeper inside the sample and allows probing of deeply buried interfaces. Consequently, the minimum sample preparation and the non-invasive approach^1^ leaves the sample intact for a possible complementary investigation with the above-mentioned microscopy. The use of sample environment (liquid, gas, high pressure and/or temperature) is also possible in an XRD experiment and rather difficult in electron-based microscopies, for which mostly high- and ultra-high-vacuum is required. Acquisition of data under extreme (pressure, temperature) or ‘real’ conditions (catalysis at 1 atm. pressure) thus need the use of X-rays.

X-ray spectroscopies do present some of the above-mentioned advantages and can be used in combination with XRD; this is, however, beyond the scope of the present paper.

In the following, we introduce the basic principles of XRD imaging. For diffraction experiments the material (sample) to be investigated is implicitly supposed to be crystalline (atomic long-range order), since the lattice parameter or lattice spacing is probed. Accessing the lattice parameter of the crystalline material is done *via* the Bragg law (Als-Nielsen & McMorrow, 2001 ▶; Warren, 1990 ▶),
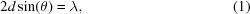
with *d* being the probed lattice spacing, θ the incident (Bragg) angle, λ the wavelength of the X-ray radiation used and *n* being an integer. It can thus probe lattice variations of Δ*d*/*d* ≃ 10^−4^, that is by far better than microscopy methods.

Of course, the method presents drawbacks as well, the main one being the work in the so-called ‘reciprocal space’ or Fourier transform space (Warren, 1990 ▶; Als-Nielsen & McMorrow, 2001 ▶). Diffraction conditions (θ angles) for families of crystalline planes (identified by their *HKL* Miller index, of lattice spacing *d*_*hkl*_) are translated into Bragg peaks (points) in the *HKL* reciprocal space. This is much less straightforward or intuitive to understand than a ‘real’ image of the sample. Also, the information extracted from XRD data originates from all the illuminated sample, the result being the incoherent sum of intensities corresponding to individual regions or objects. In a standard XRD experiment (beam size ≃ 0.1–1 mm) it is not always straightforward to disentangle the contributions coming from the different parts of the sample: if distributions can be, in general, extracted, measurements on more complicated samples (*e.g.* multi-modal growth modes) are difficult to interpret. A microscopy-like approach in such cases becomes highly desirable.

We will show here that XRD can be used as well to yield contrast, be it in full-field imaging (topography) with a high-resolution area detector or in a point-probe scanning mode approach using a micrometre-sized X-ray beam. We will highlight the advantages and the specificity of each of these methods. The paper is organized as follows: after detailing the samples used for this study, the experimental set-up will be described for each type of measurement and then the obtained results are detailed. The first experimental approach uses X-ray diffraction contrast in a full-field imaging mode at the Bragg angle (topography) in order to obtain information about the sample and the possible presence of defects. The reader can find details about the technique in the work of Moore (1995 ▶, 2009 ▶, 2012 ▶), Moore *et al.* (1999 ▶), Bowen & Tanner (1998 ▶), Yacoot *et al.* (1998 ▶), Wierzchowski & Moore (2007 ▶) and Hoszowska *et al.* (2001*a* ▶,*b* ▶) (and references therein). The second approach (microbeam X-ray diffraction) is used to access local information about the crystallinity of the sample and evidence inhomogeneities in crystalline structure, close to the edges of the lithographed objects at micrometre length scale.

Well established methods which could evidence such features exist and are used at a number of synchrotrons worldwide (HASYLAB, APS, ESRF). We can point here to the use of polychromatic-focused X-ray beams [Laue microbeam diffraction (Budai *et al.*, 2003 ▶; Larson *et al.*, 2002 ▶; Chung & Ice, 1999 ▶; Ice & Larson, 2000 ▶; MacDowell *et al.*, 2001 ▶)] or tracking procedures [combining ‘the monochromatic rotation method with X-ray tracing’ in order to access dis­locations, strain and lattice rotation of individual grains (Poulsen *et al.*, 1997 ▶, 2001 ▶; Lauridsen *et al.*, 2001 ▶; Margulies *et al.*, 2001 ▶, 2002 ▶; Poulsen, 2004 ▶; Martins *et al.*, 2004 ▶; Jakobsen *et al.*, 2006 ▶; Winther *et al.*, 2004 ▶; Schmidt *et al.*, 2004 ▶)]. Similar effects (for semiconductor samples) were shown in an approach developed by Murray *et al.* (2003 ▶, 2005 ▶, 2008 ▶, 2011 ▶): they show not only that lattice spacing selectivity is achieved but also that lattice plane rotations are shown as well and quantified with sub-micrometre lateral resolution. This last cited method is thus more adapted for the type of systems investigated in this paper, as a complementary technique to the Bragg topography.

## 2. Samples   

The model samples used for this study are metal–oxide tunnel junctions made by optical lithography. In order to investigate possible morphological anisotropy effects we prepared objects with sizes ranging from 150 µm down to 10 µm and various shapes: discs, squares and rectangles. Magnetic tunnel junctions (two ferromagnetic electrodes, a hard and a soft magnetic layer, separated by an insulating layer) have been thoroughly studied over the past years because of their applications in spintronic devices at an industrial level. A number of fundamental issues regarding these structures, as well as for other multilayered systems, are still unresolved. For various layer compositions and preparation conditions, the tunnel magneto-resistive response has been extensively investigated and correlated to the (crystalline) structure and interfacial features (De Teresa *et al.*, 1999 ▶), by techniques like transmission electron microscopy (TEM), spectroscopy or X-ray diffraction. However, the effect of the lithography process itself, especially when the junction size, or more generally the size of the lithographed object, decreases, started to be investigated only recently (Murray *et al.*, 2003 ▶, 2005 ▶, 2008 ▶, 2011 ▶; Mocuta *et al.*, 2007 ▶, 2009 ▶).

The samples consisted of multilayers made of epitaxial metal and oxide layers of 15 nm Co/1.5 nm γ-Al_2_O_3_/5 nm CoFe_2_O_4_ and 15 nm Co/3 nm γ-Al_2_O_3_/25 nm Fe_3_O_4_ grown on α-Al_2_O_3_(0001) substrate. Two different thicknesses (0.3 mm and 1 mm; see further text for details) for the α-Al_2_O_3_(0001) substrate were used, the former thickness providing better thermal conductivity and electrical charge dissipation. A 10 nm-thick Pt buffer [known to increase the crystalline quality of the subsequent metal oxide layers (Barbier *et al.*, 2005 ▶, 2007 ▶)] was epitaxially deposited on the substrate prior to the metal–oxide layer growth. The oxide layers were epitaxially grown by atomic-oxygen-assisted molecular beam epitaxy (AO-MBE) (Gao & Chambers, 1997 ▶; Moussy *et al.*, 2004 ▶; Gota *et al.*, 1999 ▶, 2000 ▶) and had very good crystalline quality, as checked by high-resolution TEM (not shown here; see Mocuta *et al.*, 2009 ▶) and XRD (see later in this paper). A 15 nm Au capping layer was applied for protection against oxidation. The crystalline quality of the layers and the sharpness of the different interfaces was checked during the growth by *in situ* reflection high-energy electron diffraction (RHEED) (Mocuta *et al.*, 2009 ▶) and *ex situ* by cross-sectional high-resolution TEM (Moussy *et al.*, 2004 ▶; Ramos *et al.*, 2007 ▶).

The optical lithography process was performed *ex situ* (Bataille, 2005 ▶; Nassar, 1999 ▶; Bowen *et al.*, 2003 ▶) on a specially modified lithography machine (water-cooled sample holder and use of neutral Ar atoms instead of Ar^+^ ions) (Bowen, 2003 ▶; Bowen *et al.*, 2003 ▶). Owing to the presence of the different layers, the realisation of a tunnel junction structure requires several lithography steps:

(i) Definition of the junctions and etching of the upper (free) layer;

(ii) Definition of the lower tracks (Pt electrode) and etching down to the substrate, through the different layers (free layer, tunnel barrier, pinned layer);

(iii) Encapsulating the whole sample into Si_3_N_4_ insulator;

(iv) Opening the insulating layer to access and contact the upper and lower electrodes;

(v) Making the contacts.

**Figure 1:**
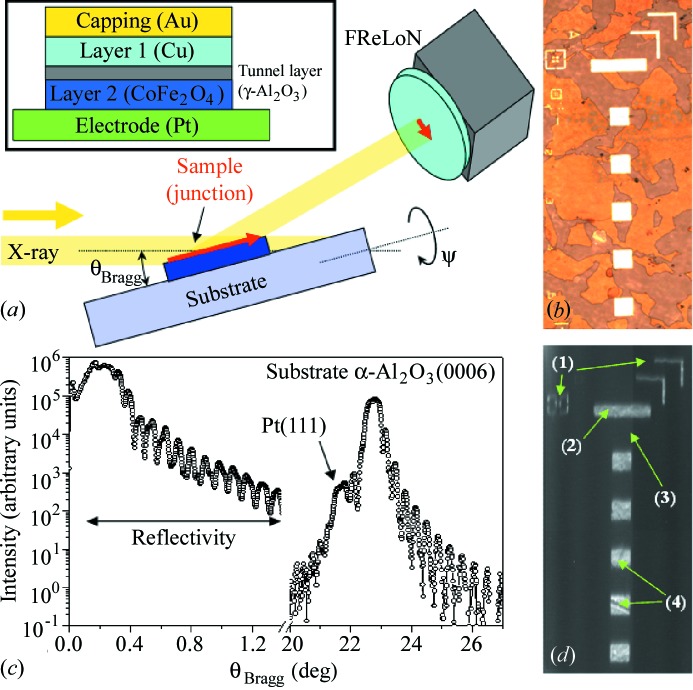
(Colour online) (*a*) Schematics of the diffraction imaging experiment. The sample is illuminated by a parallel X-ray beam under an incident angle set to the Bragg angle (θ_Bragg_) of the Pt(111) buffer layer. The elevation angle (eulerian geometry) is ψ. The sample image is recorded by the high-resolution area detector (FReLoN). The part of the sample diffracting (Pt) is the part labelled as ‘electrode’ [the inset shows the multi-layered structure of the sample (tunnel junction)]. (*b*) Optical microscopy image of the 120 µm-sized square junctions. (*c*) Radial scan (*i.e.* θ–2θ geometry) performed for a thick sample using a point detector (*E* = 15 keV; pink beam: Δ*E*/*E* = 10^−2^). (*d*) Images of the tunnel junction area taken for an incident angle of the sample set as the Bragg angle of the Pt peak; (1) mask alignment marks for lithography; (2) contact electrode; (3) junction area of the FReLoN chips; (4) square-shaped junctions (some of them with defects appearing as scratches).

The precise alignment of the masks between the different steps, as well as an accurate control of the etching processes for the different layers, is mandatory to guarantee the proper columnar structure shown in the inset of Fig. 1(*a*) ▶. The last two steps are necessary for a measurement of the magnetic behaviour of the device (tunnel junction) but were not carried out in the case of the samples used for the *ex situ* XRD measurements.

Object sizes from 10 to 150 µm lateral size and various shapes (squares, rectangles and discs) were made on the same sample. The average distance between the different objects (0.5 mm) is large enough to separate them well as isolated objects when using beam sizes of about a few 100 µm. The higher lateral resolution imaging and focused X-ray beams are then needed to access local details (defects) in the junctions.

Interestingly, often for the XRD measurements, there is no need to remove from the sample surface the resist used during the lithography process. Figs. 1(*b*) ▶ and 3 (top panels) show optical images of the lithographed samples with the resist still present on the samples surface (crackled dark zones). Indeed, at the rather high energies used (≥7 keV), the X-rays cross the resist layer and suffer low absorption; the non-crystalline resist will simply not scatter (diffract) in regions close to the signal originating from the metallic layers.

## 3. Experimental set-up   

Two experimental approaches using the diffraction signal in order to obtain a contrast are described in this section. Their complementarity will be discussed in §4. The experiments reported here were performed at beamlines BM-05 and ID-01 at the European Synchrotron Radiation Facility (ESRF) in Grenoble, France. On beamline BM-05, both pink-unfocused and monochromatic (parallel and focused to micrometre size) beams were used, while the ID-01 measurements were performed only in monochromatic non-focused (parallel) beam.

### 3.1. Full-field imaging with diffraction contrast   

The experimental set-up is depicted in Fig. 1(*a*) ▶. For a detailed description of the technique and the experimental approach, the reader is referred to Moore (1995 ▶, 2009 ▶), Moore *et al.* (1999 ▶), Bowen & Tanner (1998 ▶), Yacoot *et al.* (1998 ▶), Wierzchowski & Moore (2007 ▶) and Hoszowska *et al.* (2001*a* ▶,*b* ▶) (and references therein). The sample (CoFe_2_O_4_ oxide layer, 1 mm-thick α-Al_2_O_3_ substrate) containing the lithographed tunnel junctions is placed on the diffractometer; a parallel X-ray beam illuminates its whole surface (or at least the big junctions of interest, of sizes ∼100 µm). The sample is mounted with one of its edges parallel to the X-ray beam. An optical microscope points to the centre of rotation of all the circles of the diffractometer (centre of the confusion sphere) and allows a pre-positioning of the sample (and of the region of interest) in the X-ray beam. A Fast REadout LOw Noise (FReLoN 2000) CCD camera (http://www.esrf.eu/Users AndScience/Experiments/Imaging/ID22/BeamlineManual/Detectors/Cccd/Frelon) with a pixel size of 0.7 µm and a two-pixel point-spread function was used^2^ (Labiche *et al.*, 2007 ▶; Coan *et al.*, 2006 ▶). A pink beam (multilayer monochromator, energy resolution Δ*E*/*E* = 10^−2^) at an energy *E* = 15 keV was used in order to increase the available flux (Ziegler *et al.*, 2004 ▶). Although a poorer angular resolution is expected as a consequence of the degraded energy resolution, it still remains good enough for the investigated system, for which very thin layers (in the several 10 nm range) are measured (see Appendices *A* [App appa] and *B* [App appb]).

In a first approach an X-ray reference alignment is performed; this can be realised using a point detector as well as an area detector. The sample surface is aligned in the X-ray beam, then specular reflectivity and radial scans are performed [the momentum transfer vector **q** is kept all the time perpendicular to the sample surface, *i.e.* the incident angle is kept equal to half of the detector angle, θ–2θ geometry, Fig. 1(*c*) ▶]. The small θ angle regime of the reflectivity curve presents characteristic Kiessig fringes; these oscillations have as origin the well defined thickness of the layers (and interference between them) and are easily identified even with the increased width due to the energy spread mentioned above. The values obtained are in agreement with the thickness of the layers deposited.

At larger θ angles the Bragg diffraction regime is accessed. We note the presence of the intense α-Al_2_O_3_(0006) substrate peak (θ ≃ 22.5°) of thickness oscillations and a shoulder (θ ≃ 21.6°) attributed to the CoFe_2_O_4_(111) and Pt(111) Bragg peaks, which can hardly be separated due to the degraded energy resolution. The effect of this poorer energy resolution also has the effect of broadening the substrate peak and smearing the signal (oscillations) (see discussion in Appendix *A* [App appa]).

After this preliminary alignment procedure, the full-field imaging approach can be started. The incident angle for the sample is set to the Bragg angle of the Pt(111) and the area detector is positioned at the corresponding Bragg angle (2θ = 43.2°). Full-field images of the sample are recorded by the area detector. The contrast which will be used later is extracted by recording the diffracted beam.

The same approach was also used to investigate the 0.3 mm-thick substrate sample. The results are detailed in §4.1.

### 3.2. Raster imaging using focused X-ray beams as local probe with diffraction contrast   

The second approach we will show here makes use of a focused X-ray beam (micrometre size) and raster scanning the sample in this beam, while recording diffraction patterns (diffraction contrast). The approach has been detailed by Mocuta *et al.* (2008 ▶) and Stangl *et al.* (2009 ▶). The following is a brief review of the technique.

A monochromatic X-ray beam (Δ*E*/*E* ≃ 10^−4^) of energy *E* = 7 keV is used. The X-rays are focused using 39 beryllium compound refractive lenses (Be-CRLs) (Lengeler *et al.*, 1999 ▶; Snigirev *et al.*, 1996 ▶). The resulting spot size was 3.2 µm × 7 µm (vertical × horizontal, full width at half-maximum, FWHM) with a measured photon flux in the spot of 10^8^ photons s^−1^. The measurements are performed typically at an incident angle ∼30°; thus the beam footprint on the sample can be considered as disc-shaped with a 6–7 µm diameter.

The diffractometer angles (sample incidence and detector angles) are set to fulfil Bragg equation (1) for lattice planes corresponding to the various present crystalline structures (layers). In this way, diffraction contrast of individual layers is obtained. Then, without changing the diffractometer angles, the sample lateral position is scanned in the X-ray focused beam. The scattered intensity measured by the detector^3^ is recorded for each lateral point position on the sample. The result is a raster image of the sample corresponding to the chosen layer. By tuning the diffraction angle to be characteristic of the crystalline structure of each layer, a different image is obtained [see, for example, Fig. 4 (left panel)].

Since the Be-CRLs are mounted on a translation stage operating perpendicularly to the X-ray beam, it is possible to easily shift them in and out of the beam, thus switching from a microbeam configuration to one in which a flat and parallel monochromatic beam is illuminating the full sample. The accuracy of the translation stage ensures a good reproducibility in position and size of the focused X-ray beam.

It is thus rather easy to switch from a full-field to a raster microbeam imaging configuration. If the former allows a quick identification and ensemble view of the sample (or of large regions of it), the latter will allow more detailed characterization of the defects at the micrometre scale. This combination of the two imaging modes is shown hereafter in §4.

## 4. Results and discussion   

The effect of optical lithography on the different epitaxial layers will be addressed here using local probe XRD; we will discuss the effects of substrate thickness, size and shape of the junctions.

### 4.1. Full-field imaging in pink beam   

A 15 nm Co/1.5 nm γ-Al_2_O_3_/5 nm CoFe_2_O_4_ sample deposited on a 1 mm-thick substrate is considered first. The measurements in θ–2θ geometry (Fig. 1*c* ▶) allow to extract the thickness of the layer from reflectivity measurements but also to determine the angular positions corresponding to the different Bragg peaks characteristic for the various layers of the sample. The most intense one seems to be the Pt(111) + CoFe_2_O_4_(111).^4^

Let us now look at the full-field image. The results measured for the Pt(111) Bragg position are shown in Fig. 1(*d*) ▶, and are compared with an optical image of the sample. The same region is shown in Fig. 1(*b*) ▶. 16 images of 30 s exposure each were summed to produce the resulting image shown in Fig. 1(*d*) ▶. The resulting image (topography) is corrected by the projection due to the incident angle θ. Indeed, it can be seen in Fig. 1(*a*) ▶ that the size of the object (red arrow) imaged on the area detector has to be divided by the sin(θ) factor in order to obtain its real size. With only this correction and by knowing the pixel size on the camera (from calibration or camera characteristics), the image obtained matches perfectly with the corresponding optical image of the sample.

**Figure 2:**
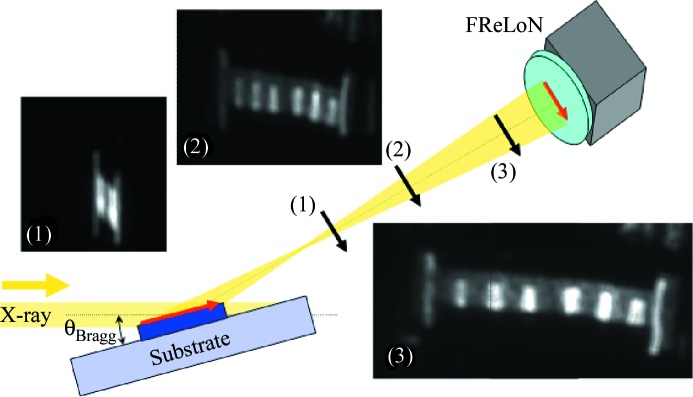
(Colour online) Schematics of the full-field imaging experiment (the case of the bend sample, 0.3 mm-thick substrate). The parallel incident X-ray beam is focused by the sample, both in the longitudinal and sagittal directions. The result is a distorted image on the area detector. Images at several distances are taken: (1) close to focal point; (2) out of the focal point and (3) far away from the focal point. The structure of the sample is similar to the one shown in Fig. 1(*b*) ▶.

We should note here the presence of some scratch-like features in Fig. 1(*d*) ▶, features labelled as (4), although no corresponding defect is seen in the optical image (Fig. 1*b* ▶). This can be understood in terms of the probed signal in the two images. In the optical image the morphology of the surface of the sample is essentially probed by the amount of reflected light. It is indeed expected that the surface quality of the deposited layers (and consequently of the objects after lithography) is very good. In the XRD image, the diffracted intensity, coming from micrometre-deep layers, is recorded. Most probably the darker area corresponds to a zone in which a crystalline defect appeared (*e.g.* grain boundary). This behaviour is similar to that seen by Hoszowska *et al.* (2001*a* ▶,*b* ▶). A similar sample (0.3 mm-thick substrate) was then investigated using the full-field XRD contrast. Surprisingly, the first obtained images could barely be correlated with the corresponding optical images of the sample surface (Fig. 2 ▶). By taking images with the area detector placed at different distances from the sample, a magnification effect of the image could be identified.

We understood the different images as the result of X-ray focusing by the sample itself, which is confirmed by the ‘zoom effect’ visible when going with the area detector further away from the sample. The measurements made at different distances allowed the macroscopic curvature of the sample (sagittal and longitudinal directions) to be determined.

We can note that the images are distorted both along the impinging X-ray beam direction as well as in the direction perpendicular to it. By knowing precisely the sample–detector distances, the size of each image (calibrated pixel size) and the size of the illuminated junctions, one can deduce that the shape of the sample is concave, with radius of curvature values of ∼1.8 ± 0.3 m and 2.7 ± 0.4 m in the sagittal and longitudinal directions, respectively. These values compare well with the optical measurements of the sample curvature, which yield values of 1.4 ± 0.1 m and 3.0 ± 0.5 m, respectively. The optical measurement confirmed as well that the curvature directions (astigmatism) were only 3° off the edges of the rectangular sample (*i.e.* from the X-ray beam direction illuminating the sample in the XRD experiments).

**Figure 3:**
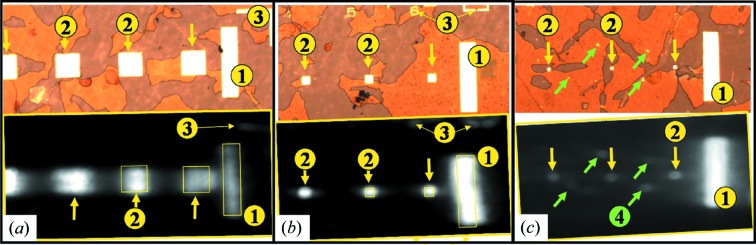
(Colour online) Two-dimensional images of an array of square junctions [from (*a*) to (*c*), 120, 30 and 10 µm size, respectively] (top = optical image, bottom = area detector image). The images were recorded far from the focal point. The different parts of the sample are identified as follows: (1) external contacts; (2) square junctions; (3) alignment marks used in lithography; (4) parts of the film not removed during the lithography. Images were corrected by the projection due to the Bragg angle and focusing effect (sample curvature).

Once this ‘zoom’ effect is known, it can be used in order to *enhance* the resolution achieved during the measurement, similar to imaging modes with high-divergent X-ray beams (see, for example, Rau *et al.*, 2006 ▶; Rau & Liu, 2007 ▶). Knowing the sample radius of curvature, the full-field images can be corrected. In order to explore and test the achievable resolution, images of various areas of the 0.3 mm-thick sample were recorded with the area detector placed far away from the sample (two to three times the focusing distance given by the bend sample, as found in Fig. 3 ▶). Fig. 3 ▶ shows a comparison of optical^5^ and full-field images for square junctions of lateral sizes of 120 µm, 30 µm and 10 µm. The full-field images were corrected by the incident angle θ and the measured radius of curvature; the results compare well with the corresponding optical images. Various parts of the sample are highlighted: the square junctions (2), the alignment marks (3) and the contact pads (1). All the sizes down to 10 µm can be identified. Moreover the presence of defects in lithography are highlighted in Fig. 3(*c*) ▶: the bright spots seen in the optical image [Fig. 3(*c*) ▶, top] are metallic structures. By tuning the diffraction angle to be sensitive to various layers, one can determine whether the layer structure is intact or whether some of the layers were etched away.

One may note here that the resolution of the area detector and of the whole set-up is good enough to see details like the alignment marks or defects on the objects. This method can be used also as a first approach in finding the objects of interest before switching to the focused beam experiment (see §3.2, §4.2 and §4.3).

### 4.2. Raster imaging in monochromatic focused X-ray beam   

The power of X-ray diffraction using microbeams (µXRD) in a two-dimensional scanning approach (raster maps) can be illustrated in the following example. A particular lithographed sample containing several junctions of various sizes was considered. The layer structure of the sample (15 nm Co/3 nm γ-Al_2_O_3_/25 nm Fe_3_O_4_) was slightly different from the structure of the other samples used for this study: the hard magnetic oxide layer ‘layer 2’ (typically CoFe_2_O_4_) was replaced by Fe_3_O_4_. This particular sample, which from the point of view of lithography can be considered as a ‘bad’ sample, was in fact obtained during checking and validating alignment procedures between the various steps in the lithography process. The result is that the different etching regions are shifted, resulting in a shift of the mask corresponding to the different layers/objects, as can already be seen in an optical image of the sample. For XRD, the result is a heterogeneous sample with laterally shifted areas having different diffraction signals, as will be detailed in the following.

**Figure 4:**
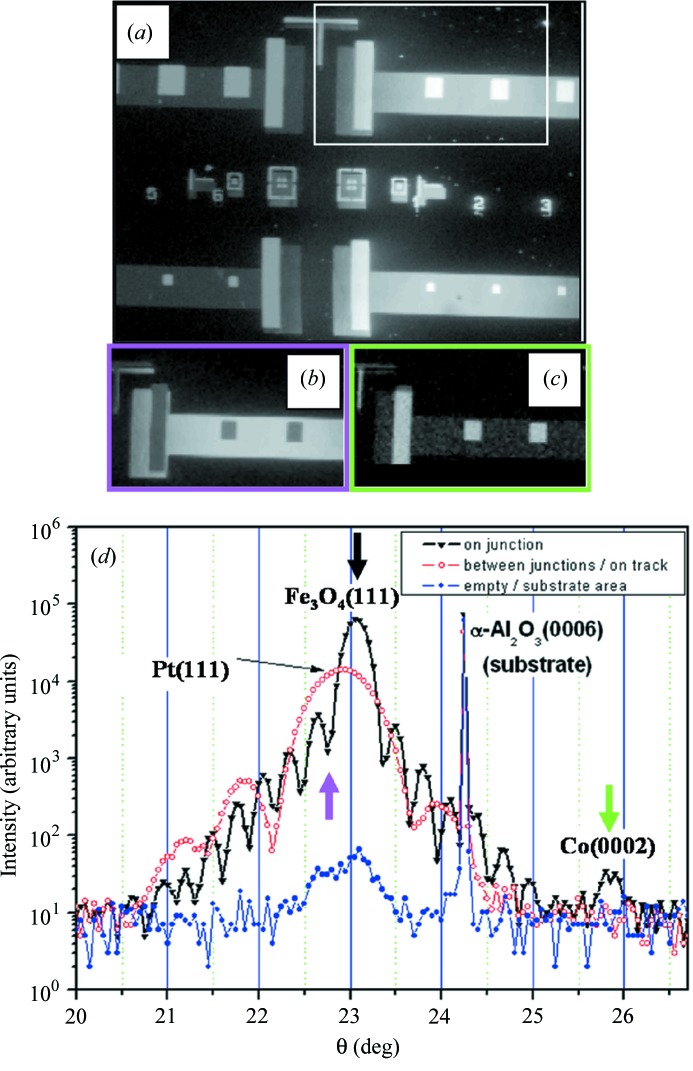
(Colour online) A poorly lithographed sample is used. (*a*, *b* and *c*) Several raster maps with diffraction contrast corresponding to Fe_3_O_4_, Pt and Co, respectively; the colour of the frame corresponds to the position on the radial scan (*d*) indicated by the corresponding arrow (see text for more details). (*d*) Radial scan in the vicinity of the Bragg peaks characteristic of the different layers performed with a point detector in a monochromatic (7 keV) focused X-ray beam, at different lateral positions on the sample (see inset legend). Positions in the reciprocal space (angular values) at which raster maps (*a*) to (*c*) were performed are marked by coloured arrows.

In investigating this sample, the first XRD measurement was performed on the whole sample with a large (0.1–1 mm-sized) X-ray beam. This allowed the presence of Bragg peaks originating from the different materials (Co, Pt, Fe_3_O_4_) to be identified and confirmed. Once the diffraction angles are known, raster mapping of the sample by using diffraction contrast characteristic of these layers was performed. The result is shown in Figs. 4(*a*)–4(*c*) ▶.

The image exhibits various contrast mechanisms. The patterns and contours observed in diffraction contrast maps can be easily found in agreement with the optical image of the sample (and the template of the mask). Let us discuss in detail these contrasts and how we can understand them.

(i) The image in Fig. 4(*a*) ▶ was recorded at the position characteristic of the Pt(111) (lower electrode) and Fe_3_O_4_(111) Bragg peaks (in fact these two peaks are rather close and cannot be resolved easily within the present approach). In raster imaging when using this contrast, it should thus mainly render the full mask, including the lower electrode track connecting the junctions. Apart from the misalignment, the contrast is expected at first to be uniform, possibly more intense on the junctions (owing to the extra contribution of the oxide layer). It can indeed be seen that the junctions do exhibit a higher-intensity signal. The slightly lower intensity on the left side of Fig. 4(*a*) ▶ is most probably due to a small misalignment of the sample angles when this one is laterally translated (over several millimetres) in order to perform the raster map.

(ii) The image in Fig. 4(*b*) ▶ was recorded at a slighter lower θ angle, thus closer to the theoretical position of the Pt(111) Bragg peak. The Pt is expected to exist only on the track and the junctions, thus no contrast is expected from the track and junctions. However, during the experiment a contrast is found with a *lower* intensity at the junction positions.

(iii) The image in Fig. 4(*c*) ▶ was recorded to have diffraction contrast at the Co(0002) Bragg peak. Its presence only on the junctions is indeed confirmed by the contrast measured in the diffraction raster scanning experiment. A faint and diffuse signal from the track is also present and can be used, as well, for imaging. The origin of this faint signal can be multiple: either some very small quantity of Co might still be present on the track after the lithography, or scattered signal can be detected, the signal originating from the thickness oscillations (Kiessig fringes) extending further away in angular range, close to the value corresponding to the characteristic position of the Co(0002) Bragg peak. Although the first hypothesis seems less probable (the lithography has to be performed down to the Pt layer, through the Fe_3_O_4_ layer below as well), our data do not allow it to be completely excluded. We point out here that the lower threshold of the intensity contrast scale used in Fig. 4(*c*) ▶ was set very close to the background level [7–8 counts s^−1^, see Fig. 4(*d*) ▶].

If the third case above (and possibly the first) is rather logical, how do we understand the peculiar behaviour close to the Pt(111) peak? In order to obtain a clearer image, θ–2θ XRD measurements were performed using the X-ray focused beam at different positions on the sample: on the square junction, on the track connecting two junctions and on the bare substrate (Fig. 4*b* ▶). Let us discuss these cases one by one.

(i) On the bare substrate, only the α-Al_2_O_3_(0006) peak of the substrate can be measured. Some small contribution close to the Pt(111) position can be detected (but several orders of magnitude lower than other situations). It could be attributed either to traces of Pt after the lithography process or, more probably, to the presence of a very low intensity halo around the microbeam.

(ii) On the track between junctions, in addition to the substrate peaks, only the Pt layer should exist. Its Bragg characteristic peak exhibits thickness oscillations showing its good quality. The thickness obtained through this measurement is in good agreement with the expected thickness of 10 nm.

(iii) On the junction, all the layers should exist. In the probed angular region, the 10 nm Pt(111) and 25 nm Fe_3_O_4_(111) peaks interfere due to the presence of only one high intense peak [slightly shifted towards the Fe_3_O_4_(111) expected position]. Again, thickness oscillations appear.

With this information we can now understand the contrast in Figs. 4(*a*)–4(*c*) ▶. Owing to the proximity effect of the Bragg peaks of Pt and Fe_3_O_4_, measuring at this position yields an enhancement of the signal on the junction. The broad Pt peak also produces a contrast when the track between the junctions is measured. Owing to the presence of thickness oscillations (Kiessig fringes) as mentioned in §3.1 and Fig. 4(*d*) ▶, when the XRD contrast is tuned by shifting towards the Pt position [pink arrow in Fig. 4(*d*) ▶], it might happen that we probe a node of intensity on the θ–2θ curve. The detected signal is thus lower than the corresponding intensity measured at the maximum of the Pt(111) + Fe_3_O_4_(111) peaks. This is exactly what happens when the X-ray beam is laterally positioned on a tunnel junction, and it explains well the reversed contrast obtained in Fig. 4(*b*) ▶.

This reversed contrast is, in this case, a simple interference effect. These remarks do show that we are not only sensitive to lattice parameters but, in a more general manner, to diffracted (or scattered) signals including interference effects. The information that can be obtained can be much more complex than just a lattice parameter (obtained locally, with lateral resolution).

We can also see in Fig. 4(*d*) ▶ that the thickness measured on the junction is approximately four times larger than that found on the track (the period of the oscillations is about four times smaller). This is in agreement with a total thickness of the Pt and Fe_3_O_4_ layers (35 nm) which is about four times larger than the thickness of the Pt layer (10 nm).

### 4.3. µXRD experiments: local defects   

With an X-ray microbeam scanning approach, it is possible to show (crystalline) defects inside the junctions (either being sensitive to the crystallinity *via* the lattice parameter or to the layer thickness using thickness oscillations). In the following, the crystalline defects close to the objects edges were measured.

We have shown in the previous section how the micrometre-sized X-ray beam can be used for local probe imaging with diffraction contrast. Besides this imaging approach, a high-resolution XRD study with lateral resolution (essentially given by the spot size) is also possible. Some results covering this topic were already shown above (Fig. 4*d* ▶), in which the crystalline structure and thickness of various areas of the sample were accessed.

We will show here a second example of measuring locally crystalline defects in these samples. The approach was introduced by Murray *et al.* (2003 ▶, 2005 ▶, 2008 ▶, 2011 ▶; Mocuta *et al.*, 2007 ▶, 2009 ▶). The sample used in this case is one having CoFe_2_O_4_ as a pinned layer and with the lithography process completed correctly. High-resolution XRD data are acquired at various positions across tunnel junctions of different sizes and shapes. They allow one to determine the tilts of the crystalline planes corresponding to the different layers. If in the previously reported results only cross-section data along one direction were reported, with this experimental set-up we can also probe both directions. This is done by tuning the angular resolution of the detector (by using detector slits) either in the vertical or horizontal direction. Then the sample angles have to be scanned, either in (i) the incident angle θ, thus sensitive to crystalline tilts in the direction along the X-ray beam (longitudinal), or in (ii) the elevation angle ψ, thus sensitive to crystalline tilts in the direction perpendicular to the X-ray beam (transverse or sagittal).

In fact, in both cases, the tilts of the crystalline planes are probed inside the plane described by the scanning angle, which is in one case along the X-ray beam, and perpendicular to it in the second case.

**Figure 5:**
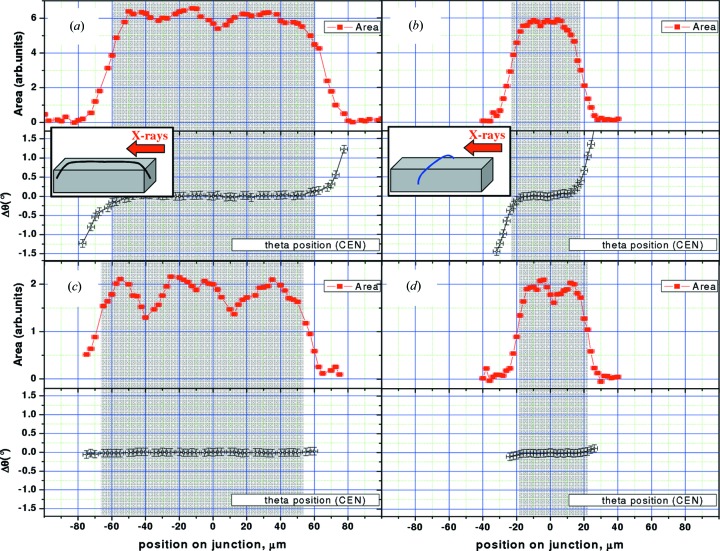
(Colour online) Crystalline plane tilt angles (Δθ) measured in a µXRD experiment for a rectangular tunnel junction (Co/Fe_3_O_4_ sample, 120 µm × 40 µm) for the Co layer (*a*, *b*) and CoFe_2_O_4_ layer (*c*, *d*). At each lateral position of the X-ray beam on the junction the integrated intensity of the peak (rocking scan) (top panels) and the position of the centre of mass of the peak (bottom panels) are reported. The lateral position is displayed on the horizontal axis with respect to the centre of the junction. The reported error bar represents the angular step used when scanning (typically 0.1–0.2°). The hatched rectangular area is the corresponding width of the junction, as from lithography parameters (120 µm and 40 µm, respectively). The insets show schematically the experimental geometry considered: the diffraction plane (containing the incident and scattered X-ray beam) is perpendicular to the surface of the sample and contains the arrow labelled ‘X-rays’. The thin line describes the lateral position of the X-ray focused beam on the sample; its curvature schematically illustrates the tilt of the Co(0001) crystalline planes.

**Figure 6:**
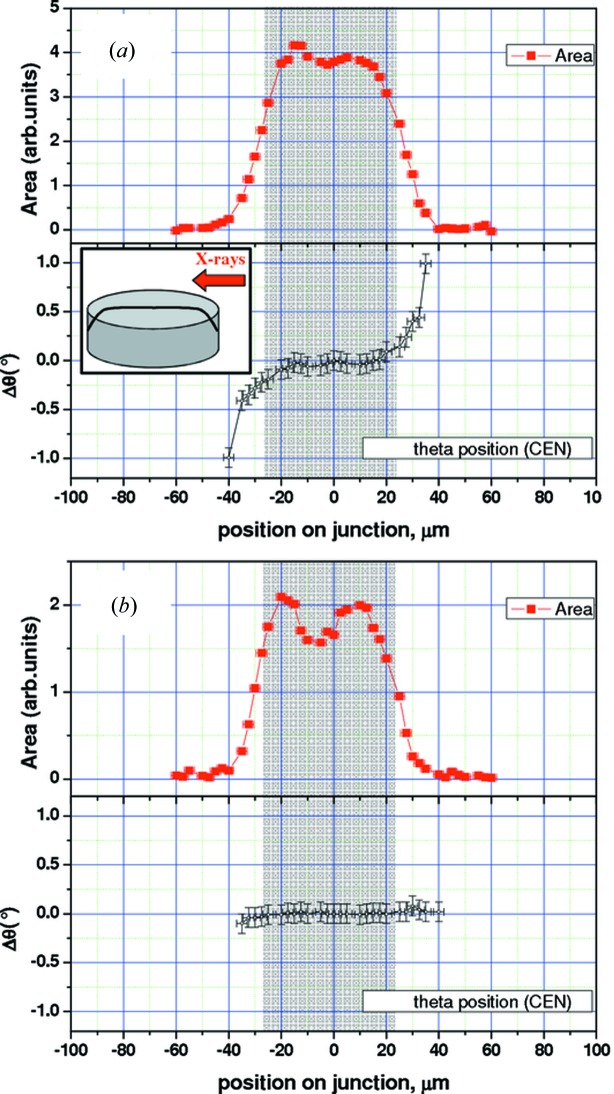
(Colour online) Similar to Fig. 5 ▶, crystalline plane tilt angles (Δθ) measured in a µXRD experiment for a 50 µm-diameter disc-shaped junction: (*a*) Co layer and (*b*) CoFe_2_O_4_ layer.

The results for these two directions are shown for two samples of different shapes: 120 µm × 40 µm rectangular-shape magnetic tunnel junction (Fig. 5 ▶) and a 50 µm-diameter disc-shaped junction (Fig. 6 ▶). By setting the 2θ angle to be sensitive to the different layers, each of them can be measured individually. We show here the results obtained for the Co and CoFe_2_O_4_ layers.

The objects of interest (junctions) can be identified using the methods presented previously in the paper. The micrometre-sized X-ray beam is moved laterally across the tunnel junction. At each lateral position the sample angles (θ and ψ) are scanned while recording with the detector the X-ray scattered signal. The angular resolution of the detector is adapted in consequence to the scanned direction (θ or ψ) by closing the detector slits [with the geometry in Fig. 1(*a*) ▶, in the vertical and horizontal directions, respectively]. The resulting curves are then fitted in order to extract the following.

(i) The integrated area beneath the curve, which corresponds to the total scattered signal from the probed layer. This area is representative of the quantity of layer in the X-ray beam and is used to determine when, or when not, the beam illuminates the junction.

(ii) The θ position of the peak. The shift between the measured value (θ_exp_) and the value (2θ/2) measured in the centre of the junction is also reported (Δθ = 2θ/2 − θ_exp_). This value corresponds to the one expected for a Co/Fe-oxide film, as measured in the centre of large lithographed features (alignment marks and contacts), and thus without, or with minimal influence from, the edges. The results are also supported by studying a Co film for a similar system (Co/Fe_2_O_3_; Bezencenet *et al.*, 2010 ▶). We show the presence of tilts of the crystalline planes with respect to the theoretical position expected for an epitaxial layer, a position which is in fact found in the centre of the junctions.

(iii) The width of the curves. A possible enlargement could give an indication about the degraded quality of the crystalline layer.

Let us now discuss the situation of the rectangular junction, shown in Fig. 5 ▶. The top curves (red) in each panel [(*a*)–(*d*)] show the integrated area (in arbitrary units) of the peak at different points, corresponding to the different positions of the X-ray beam across the junction. We have also reported the theoretical size of the junction in the corresponding direction by the shaded rectangular shape. This size is the one used for the design of the lithography mask and confirmed by optical microscopy. It can be easily seen that the agreement is very good for both Co and CoFe_2_O_4_ layers. We can also note the presence of some fluctuations in the integrated intensity value; if most of the time they are within the statistics error bar, sometimes they are significant of local defects on the sample, as the ones seen in Fig. 1(*d*) ▶ and labelled (4).

The Δθ shift angle is also reported, for lateral positions of the beam on the junction, in order to have a well defined peak (*i.e.* situations for which the integrated area of the peak is ≳10% of its maximum value, in the centre of the junction). For the Co layer, we found a tilt of the Co(0001) crystalline planes close to the edges of the junction (Mocuta *et al.*, 2007 ▶) with an amplitude up to 1–1.5°. The inset in Fig. 5 ▶ shows schematically, for each measurement geometry, the convex tilting of the measured crystalline planes. Then the CoFe_2_O_4_ layer is characterized in the same way. Similar features are found, but the amplitude of the effect is much smaller (≃0.2°) as reported in Figs. 5(*c*) and 5(*d*) ▶.

For this rectangular-shaped junction, its rather large size in both directions (>10 µm) has as a consequence a relaxation of the layers with approximately the same amplitude along both the long and short side. A lower tilt amplitude as a consequence of a reduced lateral size of the object was only noted for junctions sizes ≤10 µm (Mocuta *et al.*, 2007 ▶).

In order to check whether the shape of the tunnel junction has any effect on this tilt of the crystalline planes in regions close to the edges, a disc-like shape was measured (Fig. 6 ▶). Again a convex tilting is found for the Co planes but the amplitude of the effect is now slightly smaller, with amplitudes in the 0.5–1° range. In the case of the CoFe_2_O_4_ layer the effect seems to be present as well, again with a smaller amplitude, which is now close to the error bar of the measurement. We can understand this lower amplitude in the following way: it is expected that the layers will relax close to the object edges. This results in the tilts shown here. In the case of a square or rectangular-shaped object, the whole edge can deform, with approximately the same amplitude. For a disc shape, for each point, the nearby regions will have the tendency to deform along the radial direction, thus limiting mechanically the amplitude of this tilt. This kind of effect and similar approaches can also be found by Murray *et al.* (2003 ▶, 2005 ▶, 2008 ▶, 2011 ▶).

We should point out that the amplitude effect measured here using focused X-ray beams is *not* the macroscopic curvature, for the following reasons.

(i) It appears close to the junction edges and is not found in their centres. A macroscopic curvature will tilt the crystalline planes continuously from one edge to another.

(ii) Its amplitude (∼1° for the Co layer) is one order of magnitude larger than the one resulting from the macroscopic curvature. This last one was estimated (from the measurements) to ∼0.02–0.03° for a 200 µm junction from its centre to its edge (and ten times less for the 1 mm-thick substrate).

The crystalline quality of the epitaxial layers was found to be unchanged after lithography. The peak widths (FWHM) of the Co and CoFe_2_O_4_ layers amounted to 1.3° ± 0.3° and 0.4° ± 0.1°, respectively. These values were found for all junctions and for all measured lateral positions, and are compatible with the initial crystalline quality of the layers before the lithography process.

## 5. Conclusion   

We have shown here how diffraction contrast imaging of epitaxially magnetic tunnel junctions based on metal–oxide interfaces and made by optical lithography can be used to characterize the different individual objects and their individual constitutive layers, in evidencing the presence of local defects. Two imaging approaches were detailed: one in full-field mode, using a parallel X-ray beam and an area detector, and the other in raster scanning mode, using a micrometre-sized focused X-ray beam.

Moreover, access to crystalline defects locally, with a lateral resolution of a few micrometres (essentially given by the spot size), is shown. We observe the presence of defects on some tunnel junctions on the full-field images, defects which can be attributed to the eventual presence of dislocation lines. The examples detailed in this paper concern mostly the tilt of the crystalline planes of the layers composing the oxide/metal junction. This was probed for a disc- and a rectangular-shaped junction, in both directions (across the long and the short edge).

The morphology, crystallinity and magnetic properties for such samples are intimately related. The lithography process can modify the crystalline structure of the objects (junctions) as a whole or only close to edges. Consequently, accessing the local crystalline structure of a magnetic tunnel junction sample opens up the way to correlate these ‘defects’ with its magnetic properties, like its magneto-resistive response (De Teresa *et al.*, 1999 ▶).

## Footnotes

1 In some particular cases, soft condensed materials (*e.g.* polymers) can be altered by the X-rays, but the technique remains essentially non-destructive for most of the materials science samples.

2 The pixel size was also checked by a calibration procedure during the experiment. A value of 0.68 µm was found.

3 A point or area detector can be used; in this last case the integrated intensity over the whole detector or only part of it (a region of interest) can be used as a probe for the contrast.

4 In fact, the two peaks cannot be distinguished, especially in the low-energy resolution pink beam; see also later in the paper.

5 The presence of the resist used for lithography can be seen as dark regions on the surface of the sample.

## Appendix A Diffraction signal broadening in parallel pink beam

When using a pink beam (degraded energy resolution by using a multilayer monochromator, and not a single-crystal one, in this case Δ*E*/*E* ≃ 10^−2^), it is expected to degrade the angular resolution in diffraction measurements. Let us discuss the visibility of the thickness oscillation (be it at small or wide angles) by considering the case of a 10 nm-thick film measured at X-ray energy *E* = 15 keV in pink beam. The period of the thickness oscillations for such a 10 nm film at 15 keV (monochromatic) can be estimated as

where λ is the wavelength of the X-ray radiation, θ is the incident angle and *D* is the thickness of the layer. In the reflectivity regime θ is small, thus cos(θ) ≃ 1. The obtained angular period value is thus (Δθ)_0_ = 0.25° [see, for example, Figs. 1(*c*) ▶ and Fig. 4(*d*) ▶]. The peak width spread (when using the pink beam) can be calculated, along the specular direction, as

with (Δθ)_0_ being the oscillation period for the monochromatic (energy *E* = 15 keV) case [see equation (2)], Δ*E*/*E* = 10^−2^ is the energy resolution and θ is the incident angle around which the oscillation is seen. All the angular values above are in radians.

In the case of the reflectivity regime (*i.e.* small incident angle θ ≃ 1°), and with the above-estimated periodicity, the peak broadening amplitude [second term in equation (3)] will be ∼0.01°.

The situation changes close to the Bragg angles (wide angle regime, θ–2θ geometry, θ ≃ several 10°). In Fig. 1(*c*) ▶, one can easily identify the most intense signal as the α-Al_2_O_3_(0006) Bragg peak appearing at θ ≃ 22.5°. Using now equation (3), we expect to, and do, measure a 0.23° broad peak; this broadening originates only from the energy spread and is not seen in the case of monochromatic measurements [see, for example, the measurement performed with a monochromatic 7 keV beam, Δ*E*/*E* = 10^−4^ in Fig. 4(*d*) ▶; the substrate peak broadening in this last case would be two orders of magnitude smaller, in the few 0.001° range].

## Appendix B Diffraction signal broadening in divergent (focused) monochromatic beam

When using monochromatic beam, we have shown before that the expected peak broadening for the substrate is in the few 0.001° range. This is valid only with the assumption of a parallel (zero divergent) incident beam. The results reported in this paper for the monochromatic beam (7 keV X-ray energy) correspond to a set-up with a focused beam. Consequently, the beam impinging the sample surface is divergent. The beam divergence can be geometrically calculated from the aperture of the focusing element (Be-CRL) and the working distance (distance from lenses to sample),

In this particular case, div ≃ 0.05°. This divergent incident beam will cause a broadening of all diffraction signals measured with this geometry. In the case of the thickness oscillations originating from the layers (typical periodicity in the 0.1–0.2° range), this effect is barely visible, but it becomes important for the measuring of the α-Al_2_O_3_(0006) Bragg peak: the single-crystalline sapphire sample should exhibit a mosaicity (peak width) in the few 0.001° range.
